# A machine learning approach on whole blood immunomarkers to identify an inflammation associated psychosis onset subgroups

**DOI:** 10.1192/j.eurpsy.2023.155

**Published:** 2023-07-19

**Authors:** G. Delvecchio

**Affiliations:** Department of Neurosciences and Mental Health, IRCCS Ca’ Granda Ospedale Maggiore Policlinico, Milan, Italy

## Abstract

**Abstract:**

Psychosis onset is a transdiagnostic event that leads to a range of psychiatric disorders, which are currently diagnosed through clinical observation. Since several years, the role of immune system in the pathophysiology of psychosis has been well-recognized, showing differences from the onset to chronic phases. In this lecture, I will show the results of our recent study that tested the hypothesis of the existence of subgroups of first-episode psychosis (FEP) patients identified by distinct peripheral immunomarkers’ profiles, possibly underpinning a subgroup-specific immunopathogenesis. More in detail, I will show the results obtained by the unsupervised machine learning model that we applied to the set of 12 peripheral blood immune gene transcripts, which we recently demonstrated to classify with high accuracy a cohort of FEP patients and HC. Also, I will report the results obtained by performing post-hoc univariate analyses using selective clinical, cognitive, and brain structural phenotypes of FEP patients and HC belonging to each subgroup identified by the computational model. I will extensively discuss two key clusters identified and validated by our model: 1) a FEP cluster characterized by the high expression of inflammatory and immune-activating genes; 2) a cluster consisting of an equal number of FEP and HC subjects, which did not show a relative over or under expression of any immune marker (balanced subgroup). Therefore, in this lecture I will emphasize that our study has been the first to demonstrate the existence of a psychosis onset subgroup identified by a peculiar multivariate pattern of immunomarkers, independently of clinical features or categorical diagnosis. This is paramount as if validated in independent samples, our clustering model could enable the sample selection in clinical trials aiming to test the efficacy of novel immunomodulant or anti-inflammatory therapies tailored to the specific inflammatory subgroup of psychotic patients.

**Disclosure of Interest:**

None Declared

